# Case Report: Evaluation strategies and cognitive intervention: the case of a monovular twin child affected by selective mutism

**DOI:** 10.12688/f1000research.14014.1

**Published:** 2018-02-23

**Authors:** Micaela Capobianco, Luca Cerniglia

**Affiliations:** 1 University of Rome “La Sapienza”, Department of Psychology, via dei Marsi, Rome, 00185 , Italy; 2 International Telematic University Uninettuno, Corso Vittorio Emanuele II, 39, Rome, 00186, Italy

**Keywords:** Selective mutism; twins; assessment; intervention.

## Abstract

The present work describes the assessment process, evaluation strategies, and cognitive intervention on a 9 years old child with selective mutism (SM), a monovular twin of a child also affected by mutism. Currently, the cognitive behavioral multimodal treatment seems the most effective therapeutic approach for children diagnosed with selective mutism (Capobianco & Cerniglia, 2018). The illustrated case confirms the role of biological factors involved in mutacic disorder but also highlights the importance of environmental influences in the maintenance of the disorder with respect to relational and contextual dynamics (e.g. complicity between sisters, family relationships). The article discusses furthermore the importance of an early diagnosis as a predictor of positive treatment outcomes.

## Introduction

Selective mutism (SM) is a developmental disorder of biological and environmental etiopathogenesis. It is characterized by the child’s significant inability to speak in specific social situations (for instance at school) or in non-familiar circumstances. The clinical and research data confirm that SM is a complex etiological multifactorial disorder whose maintenance is connected with the interaction of biological (familiarity with shyness, preterm birth) ( Capobianco & Cerniglia, 2017) and environmental early factors (anxiety and overprotectiveness of the mother) ( Capobianco *et al.*, 2010; Capobianco *et al.*, 2017; Capozzi *et al*., 2017; Pizzuto & Capobianco, 2008) Analysis of the underlying thoughts and emotions in mutacic behavior reveals fear of judgment, fear of failure and being mocked, shame, depression, feelings of worthlessness or inadequacy *,*catastrophizing, and confirms the comorbidity between selective mutism and internalizing symptoms. The typical age of onset for selective mutism is between 3–6 years, even though the disorder gets usually diagnosed belatedly during the school age (around 7–8 years). It seems that the twins condition in presence of frequent associated additional biological and environmental conditions (prematurity, maternal stress, isolation) plays a crucial role in aggravating mutacic difficulties ( Capobianco & Cerniglia, 2017; Capobianco & Cerniglia, 2018). The possibility to improve and change the symptomatology of SM-diagnosed children is strongly related to the severity of the disorder and the biological environmental factors implicated in each single case. Cognitive-behavioral intervention on the child has as its purpose to change the resulting *cognitive biases* and *emotional states*, but parallel efforts on the environmental dynamics that determine the disorder maintenance are needed (family and school).

## Case report

F is a child with a belated diagnosis of selective mutism (diagnosed after the third grade of elementary school). The mutacic symptomatology appears particularly in the school environment, in interaction with teachers and peers during teaching activities. She has a monovular twin sister (L) with the same diagnosis. She is born at 36 weeks without any particular pre- or perinatal problems. The mother describes F’s development as regular through the “normal” stages and does not recall any developmental problems in the motor and/or linguistic realms during F’s first years of life ( Capobianco *et al*., 2017; Pizzuto & Capobianco, 2008).

In short, F has always been a very shy girl, showing difficulties and fear in relating to non- familiar people . F has always perceived similar situations with an intense discomfort, anxiety and concern and has learned to react through inhibition, behavioral and verbal withdrawal. As a result of functional evaluation (cognitive and projective tests), spontaneous observation and exploration of the ABC (through various simulation techniques with characters, drawings, written questions and cartoons) emerges fear of judgment, fear of failure and being mocked, being made fun of, (“everyone will laugh at me”), shame, and the fact that others might notice this feeling. Depressiveness and catastrophizing prevail over the possible consequences on others of her speaking, her perception of inadequacy and her feelings of worthlessness (“ *I am afraid to make a mistake*”, “ *I didn’t study*”, “ *I am not capable*”). F shows her discomfort via “critical-provocative” behavior especially when her sister L is present, although the twins seem to have a strong complicity: “a mute complicity” in keeping the mutism part.

*Maternal relationship*. The mother’s behavior represents an important maintenance factor of F’s mutism. The girl shows indeed an *anxious-dependent attachment style* (Pattern C) towards the mother. The mother actually appears to be anxious and worried showing a pressure attitude to make the girl speak (sometimes even punitive), and she often replaces F in her responsibilities. Another important aspect is the confrontation with F’s twin sister. The mother often tends to compare the two sisters. This creates conflict situations and a quest for attention by F, who uses various maternal affection manipulation strategies.

*Maternal thoughts and emotional states.* During the interviews, the mother shows extreme attention to maintaining a positive image in front others and a propensity to avoid feelings of shame to which she gives a meaning of failure, devaluation and humiliation. These aspects are strongly connected to her life story and particularly to experiences of abandonment and maltreatment. Her necessity not to *feel ashamed* and her focus on avoiding situations, in which she could be exposed to that risk, have an extreme importance to the mother of F and L.

The *belated diagnosis* has led F to reinforce her behavioral style such that, by now, has became habitual and an integral part of her way of being, and the basis of an equilibrium reached in the dependent relationship with her mother and her twin sister. Behaviors of passivity, lack of interest, creativity and pro-activeness, are products of the extended time in missing use of verbal language as a necessary means to develop the formation of concepts, thoughts, complex thoughts, and metacognition. Despite F’s functional evaluation, she has a normal intelligence level but her neuropsychological profile indicates poor cognitive functioning.

## Therapy

### Main objectives of individual therapy

1. To change dysfunctional thoughts of:

a) “Catastrophizing” and “Overgeneralization” compared to the consequences of speaking and the judgment of others: to speak does not necessarily provoke “negative judgments” by others. At this point it is necessary to reflect on different hypotheses of other one’s judgments and on the fact that talking can bring about different consequences that can often be more “useful” than not talking at all. Additionally the consideration of the fact that it is “normal” that we might not be liked by others, but that this does not and should not damage our personal value and capacities is considered. Moreover the reflection on shame as being a natural emotion, that can appear in particular moments, is considered. ( *emotion acceptance process*).

b) “Feelings of worthlessness” and sense of “incapacity” to speak: the promotion of autonomy and self-esteem. Not always do others laugh at what we say and if it happens that someone doesn’t share the same opinion this doesn’t mean that the content of speaking doesn’t have any value, independent from ones self-esteem and a negative or positive evaluation of contingent situations (for example interrogation, speaking in front of strangers, etc.) 

2. To understand and label emotions:

During the event simulation (to play and draw), F was asked at various times to indicate the emotion the character feels in that particular moment (by indicating the faces for each emotion or writing) with the scope to reflect carefully on the connection between events-emotions and their alternative consequences.

### Main objectives of intervention in the family environment

- Psycho-educational meetings with the parents of F and L on selective mutism disorder: F does not speak because she refuses to or throws tantrums as she feels a “discomfort” that makes her unable to speak. These meetings are mainly focused on changing the parents’ interpretation and thoughts on F’s mutacic behaviors.

- Indications on the parents’ behavior at home:

1. To adopt a neutral attitude towards the non-speaking, to neither underline it frequently as a problem nor show a punitive approach;

2. No replacement of F in her daily activities and relationships. Whenever any person asks her a question it is important to leave space for the girl and never insist that she answers verbally, nor reply on her behalf. To involve her in the conversation whilst accepting other ways of communication (gestures, drawings, written text);

 3. *Home working and autonomy promotion:* to let F gradually initiate small daily activities: for example pay at the newsstand, make a phone call, ask for information, etc.;

 4. To increase social interactions with F’s peers, if possible, separately from her sister and to create various individual spaces and locations;

 5. To avoid comparisons between the two girls. To dedicate a single and separate location for and with F.

### Main objectives of intervention in the school environment

 It is very important to have a common approach both at home as at school to enforce the effects of intervention:

Teachers behavior in the classroom: a) neutral attitude towards the non-speaking enforcing the non-verbal communication with them and between F’s peers; b) use of various disciplines (drawing, writing, open questions, multiple choice); c) promote and create activities in small groups with at least one peer with whom the girl talks to or gets along with.

### Critical analysis

Selective mutism in the developmental age is a complex problem that depends on the interaction of multiple individual and environmental issues that are interweaved in various ways in the life of the girl. Therefore there is a strong necessity to explore all life situations, to attempt to involve the school, parents and teachers and to reconcile between therapeutic objectives and demands of the various life context of the child. The main scope of intervention in the various social areas of F has collided against specific difficulties in the family-(e.g. the experiences of suffering of the mother) and school environment. It is often difficult to help parents to implement social relationships with the class or with other children. In F’s case the twin condition, the diagnosis and the belated intervention, have been important factors in the maintenance of the disorder. The cognitive intervention has a strong connection with the evaluation of thoughts and the emotions underlying the mutacic disorder. Neuropsychological and emotional evaluation through structured tests that demand verbal methods and sufficient collaboration is not easy in a condition where there is no verbal communication ( Pizzuto & Capobianco, 2008). It is necessary to use various answering procedures (e.g. written, non-verbal, drawings) whilst keeping the maximum of validity during the administration of the tests. The evaluation of beliefs, cognitive biases and the behaviors of the child are fundamental to be able to structure the treatment program and it is necessary to “create” additional functional strategies and procedures to explore these aspects in each individual child.

## Consent

Written informed consent for publication of their clinical details was obtained from the parent of the patient.
