# Supplementary material for: Case Report: Evaluation strategies and cognitive intervention: the case of a monovular twin child affected by selective mutism
Source: F1000Res. 2018 Feb 23;7:221. [Version 1] doi: 10.12688/f1000research.14014.1 (PMC5840613; doi:10.12688/f1000research.14014.1)
Supplement: Peer review history

# Peer review: Referee response for version 1

**DOI:** 10.5256/f1000research.15232.r31340

**Published:** 2018-03-12

**Authors:** Deny Menghini

**Affiliations:** 1 Neuroscience Department, Children's Hospital Bambino Gesù, Rome, Italy

**Abstract:**

- Please avoid References in the abstract.

**Introduction:**

- Please avoid self-quotations in the Introduction. For example, cite other studies on etiological factors as Krysanski VL A brief review of selective mutism literature. J Psychol. 2003 Jan; 137(1):29-40. ^1^
- Diagnostic criteria for “selective mutism” definition are needed: include Diagnostic and Statistical Manual of Mental Disorders, DSM-5; APA, 2013, p. 195.
- Cite other studies on single-cases: e.g. see Joseph PR. Selective mutism: the child who doesn’t speak at school. Pediatrics 1999; 104:308–309 ^2^
- Please check the year in Capobianco & Cernaglia (2017). In the Reference list is dated 2018.
- Add references for the comorbidity of internalizing symptoms and selective mutism: see Diliberto RA, Kearney CA. Anxiety and oppositional behavior profiles among youth with selective mutism. J Commun Disord. 2016 Jan-Feb;59:16-23. ^3^
- Please, include other studies on the relationship between twins and mutism: e.g. Albrigtsen V, Eskeland B, Mæhle M. Ties of silence--Family lived experience of selective mutism in identical twins. Clin Child Psychol Psychiatry. 2016 Apr;21(2):308-23 ^4^; Gensthaler A, Maichrowitz V, Kaess M, Ligges M, Freitag CM, Schwenck C. Selective Mutism: The Fraternal Twin of Childhood Social Phobia. Psychopathology. 2016;49(2):95-107. ^5^
- Last paragraph of the introduction: Please include references on the beneficial effect of CBT: see Walkup JT, Albano AM, Piacentini J, et al. Cognitive behavioral therapy, sertraline, or a combination in childhood anxiety. N Engl J Med 2008; 359:2753–2766. ^6^
- Details on the cognitive behavioral multimodal therapy are needed.

**Case report: **

- Please describe in details neuropsychological measures (e.g. IQ) and instruments adopted for the assessment (anxiety scales as MASC, semi-structured interview as K-sads).
- Avoid to mention “projective tests”. Projective measures are not believed scientifically valid measures and they are not adopted as standard procedures for the assessment or diagnosis in literature and guidelines.
- Avoid self-quotation in the “case report” session.
- A brief description of familial history of psychopathological disorder (especially anxiety) shoul be included.

**Results:**

- Please include a session of the main results of the present study obtained after the intervention and the measures used to verify the efficacy of the treatment.

**Discussion:**

- In the Discussion, describe the significance of present findings in light of what was already known about the topic (previous literature), and explain new understanding about the problem.
- Please, include limitations of the study.
- For example, the lack of cognitive measures and psychopathological instruments (especially anxiety) for the assessment and the post-treatment evaluation.

I have read this submission. I believe that I have an appropriate level of expertise to confirm that it is of an acceptable scientific standard.

## References

[1] KrysanskiVL: A brief review of selective mutism literature. *J Psychol*.2003;137(1) : 10.1080/00223980309600597 29-40 10.1080/00223980309600597 12661702

[2] JosephPR: Selective mutism--the child who doesn't speak at school. *Pediatrics*.1999;104(2 Pt 1) :308-9 1042901410.1542/peds.104.2.308

[3] DilibertoRAKearneyCA: Anxiety and oppositional behavior profiles among youth with selective mutism. *J Commun Disord*.59: 10.1016/j.jcomdis.2015.11.001 16-23 10.1016/j.jcomdis.2015.11.001 26625009

[4] AlbrigtsenVEskelandBMæhleM: Ties of silence--Family lived experience of selective mutism in identical twins. *Clin Child Psychol Psychiatry*.2016;21(2) : 10.1177/1359104515591225 308-23 10.1177/1359104515591225 26092264

[5] GensthalerAMaichrowitzVKaessMLiggesMFreitagCMSchwenckC: Selective Mutism: The Fraternal Twin of Childhood Social Phobia. *Psychopathology*.2016;49(2) : 10.1159/000444882 95-107 10.1159/000444882 27089281

[6] WalkupJTAlbanoAMPiacentiniJBirmaherBComptonSNSherrillJTGinsburgGSRynnMAMcCrackenJWaslickBIyengarSMarchJSKendallPC: Cognitive behavioral therapy, sertraline, or a combination in childhood anxiety. *N Engl J Med*.2008;359(26) : 10.1056/NEJMoa0804633 2753-66 10.1056/NEJMoa0804633 18974308PMC2702984

# Peer review: Referee response for version 1

**DOI:** 10.5256/f1000research.15232.r31484

**Published:** 2018-03-06

**Authors:** Marco Bozzali

**Affiliations:** 1 Clinical Imaging Science Center, Brighton and Sussex Medical School, Brighton, UK

The manuscript by Capobianco & Cerniglia investigates the selective mutism phenomenon in twins.

 The study is interesting and well written and it improves the knowledge about mutacic difficulties in children. I accept the paper with minor revision:

  

1. The Authors in the Conclusion section of the manuscript should better clarify the modalities of intervention on the two twins interaction.
2. A table with neuropsychological scores should be added to the manuscript to better clarify the patient’s cognitive profile.

I have read this submission. I believe that I have an appropriate level of expertise to confirm that it is of an acceptable scientific standard.

# Peer review: Referee response for version 1

**DOI:** 10.5256/f1000research.15232.r31146

**Published:** 2018-02-26

**Authors:** Mirco Fasolo

**Affiliations:** 1 Department of Neuroscience, Imaging and Clinical Sciences, University of Chieti-Pescara, Chieti, Italy

**26/02: This peer review report was mistakenly published with an Approved with Reservations status.  It has now been corrected to an Approved.**

 I have reviewed the Case Report  “Evaluation strategies and cognitive intervention: the case of a monovular twin child affected by selective mutism”.

 I think that the paper  is well written and offers a relevant clinical and research contribution to studies on selective mutism, a little know disorder that consists in an “inability to talk in unfamiliar contexts”.

 The case of a child monovular twin is very interesting because it allows to evidence  the link between biological and environmental factors.

 I accept the work but I think that:

- It is necessary to insert more details on the clinical case.
- The child is a twin and interaction with her sister is a though factor for  maintaining the mutacic behaviour, so, the relationships between the two sisters must be better presented.
- The cognitive-behavioural is the best approach for children with mutism. The authors can mention also others approaches for comparison (e.g. psychodynamic approach).
- Early diagnosis and surgery of selective mutism must be briefly presented.
- Some misprints are present in the text.

I have read this submission. I believe that I have an appropriate level of expertise to confirm that it is of an acceptable scientific standard.
